# Luminescence, Paramagnetic, and Electrochemical Properties of Copper Oxides-Decorated TiO_2_/Graphene Oxide Nanocomposites

**DOI:** 10.3390/ijms232314703

**Published:** 2022-11-25

**Authors:** Daniela Bala, Iulia Matei, Gabriela Ionita, Dragos-Viorel Cosma, Marcela-Corina Rosu, Maria Stanca, Carmen Gaidau, Maria Baleanu, Marian Virgolici, Ioana Stanculescu

**Affiliations:** 1 Physical Chemistry Department, Faculty of Chemistry, University of Bucharest, Regina Elisabeta, No. 4-12, 030018 Bucharest, Romania; 2 “Ilie Murgulescu” Institute of Physical Chemistry, 202 Splaiul Independentei, 060021 Bucharest, Romania; 3 National Institute for Research and Development of Isotopic and Molecular Technologies, 67–103 Donat Street, 400293 Cluj-Napoca, Romania; 4 Leather Research Department, National Institute for Textiles and Leather, Division Leather and Footwear Research Institute (ICPI), 93 Ion Minulescu Street, 031215 Bucharest, Romania; 5 Horia Hulubei National Institute of Research and Development for Physics and Nuclear Engineering, 30 Reactorului Str., 077125 Magurele, Romania

**Keywords:** TiO_2_ nanocomposites, EPR, photoluminescence, electrochemistry

## Abstract

The properties of newly synthesized Cu_2_O/CuO-decorated TiO_2_/graphene oxide (GO) nanocomposites (NC) were analyzed aiming to obtain insight into their photocatalytic behavior and their various applications, including water remediation, self-cleaning surfaces, antibacterial materials, and electrochemical sensors. The physico-chemical methods of research were photoluminescence (PL), electron paramagnetic resonance (EPR) spectroscopy, cyclic voltammetry (CV), and differential pulse voltammetry (DPV). The solid samples evidenced an EPR signal that can be attributed to the oxygen-vacancy defects and copper ions in correlation with PL results. Free radicals generated before and after UV-Vis irradiation of powders and aqueous dispersions of Cu_2_O/CuO-decorated TiO_2_/GO nanocomposites were studied by EPR spectroscopy using two spin traps, DMPO (5,5-dimethyl-1-pyrroline-N-oxide) and CPH (1-hydroxy-3-carboxy-2,2,5,5-tetramethylpyrrolidine), to highlight the formation of hydroxyl and superoxide reactive oxygen species, respectively. The electrochemical characterization of the NC modified carbon-paste electrodes (CPE) was carried out by CV and DPV. As such, modified carbon-paste electrodes were prepared by mixing carbon paste with copper oxides-decorated TiO_2_/GO nanocomposites. We have shown that GO reduces the recombination process in TiO_2_ by immediate electron transfer from excited TiO_2_ to GO sheets. The results suggest that differences in the PL, respectively, EPR data and electrochemical behavior, are due to the different copper oxides and GO content, presenting new perspectives of materials functionalization.

## 1. Introduction

Titania (TiO_2_)-based materials have attracted great scientific interest due to their physico-chemical properties and numerous applications. By UV photoexcitation, these materials are able to produce electron–hole pairs that can determine a series of consecutive reactions, most often involving the formation of radical reactive species [1].

TiO_2_ nanoparticles have shown good optical, electrical, and photocatalytic properties [2]. TiO_2_ is a substance with applications in various fields such as paints and plastics, water remediation, paper, and sensors [3,4,5]. The modification of TiO_2_ using metals, non-metals, carbon-based materials may lead to an improvement of its photocatalytic as well as photoelectrochemical activity. TiO_2_ absorbs only ultraviolet light due to its large bandgap (3.0–3.2 eV). The optical absorption performance in the visible region could be enhanced by adding copper oxides and graphene oxide (GO) to TiO_2_ nanoparticles [6,7]. Various methods of obtaining TiO_2_ and GO based nanocomposites and their optical and photocatalytic properties extensive characterization are reported [8,9,10,11,12].

The electrochemical response of TiO_2_-modified electrodes can be improved by the high conductivity of TiO_2_. Such electrodes were used for electrochemical measurements of guanine, adenine, and dopamine [13,14]. The TiO_2_ doped in the carbon paste electrode (CPE) sensor was developed to detect methyldopa in pharmaceutical samples since it presented excellent electrochemical behavior, correlated to better electrode applicability. This electrode may promote analyte electro-oxidation, increasing method sensibility [15]. Carbon-paste electrodes modified with Cu_2_O/CuO-decorated TiO_2_/graphene oxide nanocomposites may be a valuable and cheap alternative to determine compounds such as neurotransmitters in drug formulae.

The EPR measurements on solid TiO_2_ samples and on water suspensions of TiO_2_ samples evidenced the presence of an EPR signal due to the oxygen defects and/or to the presence of cooper, as well as the formation of reactive oxygen species (ROS) in suspensions. The generation of ROS (HO^•^, O_2_^•–^, singlet oxygen, etc.) in water titania suspensions recommends these systems as alternative oxidizing agents that can be used in the annihilation of water pollutants or can find antibacterial applications. In other fields such as cosmetics, titania-based materials should be carefully used in order to control their ROS activity. In this context, the importance of this study consists in highlighting the intimate relation between the composition, structure, and activity of Cu_2_O/CuO-decorated TiO_2_/graphene oxide nanocomposites by using physico-chemical methods. The significance of the work is high because the named nanocomposites may be used as advanced materials for various applications: environmental, medical textiles, self-cleaning surfaces, and electrochemical sensors.

## 2. Results and Discussion

### 2.1. Photoluminescence (PL) Data

**Figure 1:**
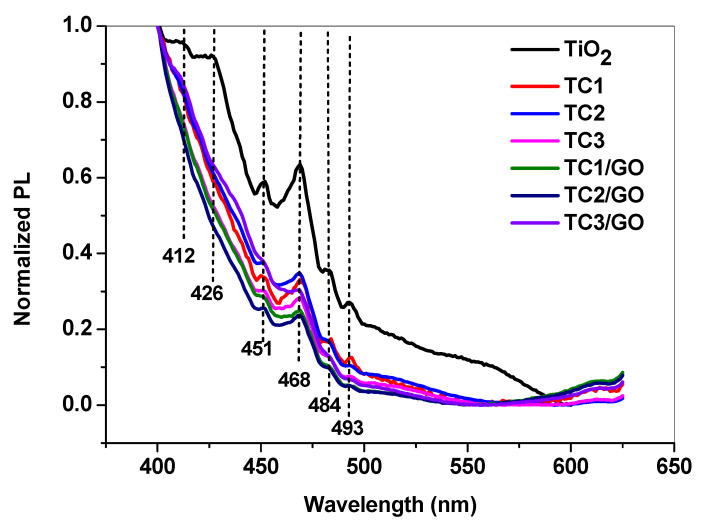
PL spectra of prepared nanocomposites compared to those of TiO_2_ nanoparticles.

As shown in Figure 1, the pure TiO_2_ nanoparticles present clear PL emission bands: at 412 nm, corresponding to the oxygen vacancy with two trapped electrons (center F) [16]; at 426 nm, attributed to the recombination of self-trapped excitons (STE) or free excitons [17,18]; at 451 nm, 468 nm, and 484 nm, assigned to electrons’ trapping in shallow traps resulting from oxygen vacancies of TiO_2_ [18]; and at 493 nm, corresponding to emissions from the TiO_2_ surface states [18]. 

As a result of TiO_2_ decoration with Cu_2_O and CuO species (identified by XPS in the previous study [9]), a lower PL intensity of TC1, TC2, and TC3 was observed, indicating an efficient charge–carrier separation. This finding is in good agreement with the data reported by M. Janczarek and E. Kowalska in their comprehensive review that presents the Cu_2_O and CuO as active species in TiO_2_ photocatalytic system being efficient electron trappers to prevent the recombination of the photogenerated electron–hole pairs [19]. This trend is more pronounced after graphene oxide addition, confirming that GO reduces the recombination process in TiO_2_ by immediate electron transfer from excited TiO_2_ to GO sheets [20,21].

### 2.2. EPR Spectroscopy Data

#### 2.2.1. EPR Spectra of Solid Samples

**Figure 2:**
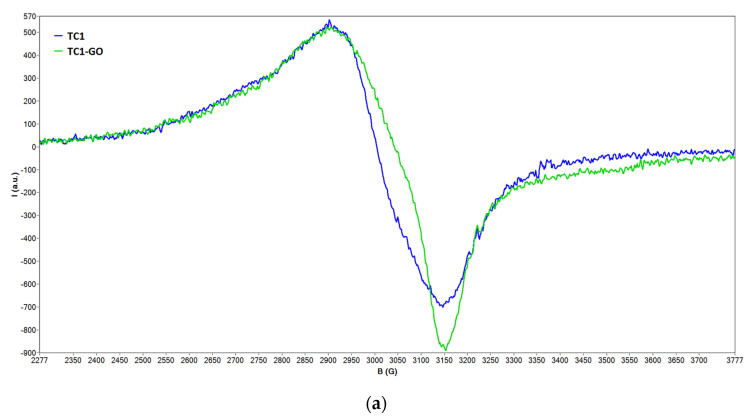

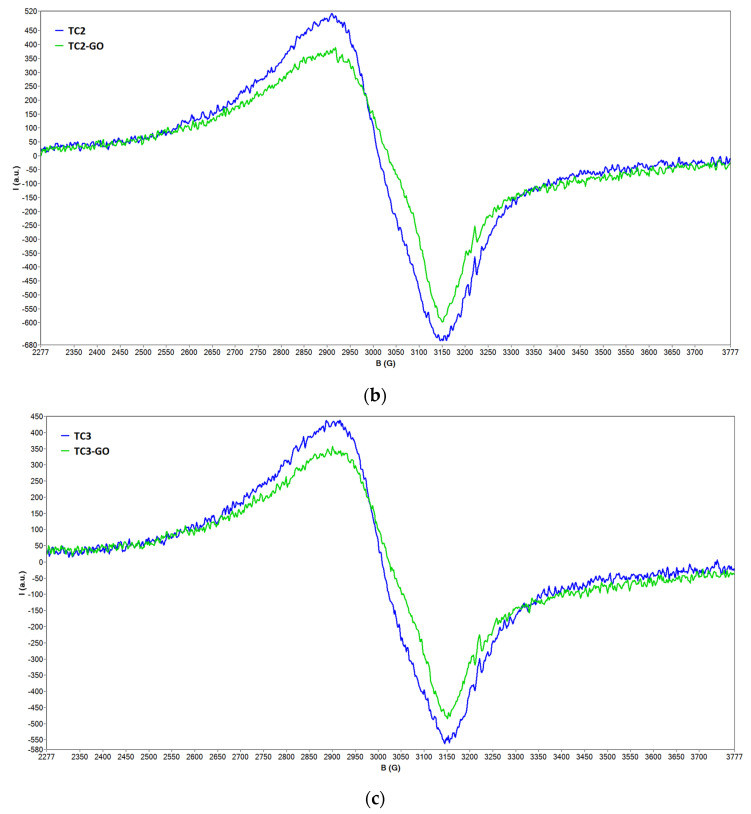
Solid state EPR spectra of the TC1/TC1-GO (**a**), TC2/TC2-GO (**b**), and TC3/TC3-GO pairs (**c**).

The EPR spectra of the solid TiO_2_ samples are presented in Figure 2. The g factors, calculated from the values of the microwave frequency (ν) and magnetic field (B) as shown in ref. [22], are given in Table 1. As can be observed from Figure 2, the EPR spectra of the copper oxides-decorated TiO_2_/graphene oxide samples present a broad line corresponding to copper (II) centers, with g factors in the range 2.1464–2.1516.

In the case of Cu_2_O/CuO-decorated TiO_2_/graphene oxide, it can be noticed that the line attributed to copper (II) becomes asymmetric, and this is due in fact to the contribution of the EPR line of the free electron due to defects present in the carbon nanomaterial and to copper (II) centers. The g values attributed to these signals are also included in Table 1 and range from 2.0810 to 2.0916. 

#### 2.2.2. Spin-Trapping Measurements

The spin-trapping method was employed in order to investigate whether the TC3 and TC3-GO samples generate ROS. Since HO^•^ and O_2_^•–^ are the radical species most commonly reported in TiO_2_ systems [11,12,23,24], two spin traps were used: 5,5-dimethyl-1-pyrroline N-oxide (DMPO), sensitive to the HO^•^ radical, and 1-hydroxy-3-carboxy-2,2,5,5-tetramethylpyrrolidine (CPH), having high affinity for the O_2_^•–^ radical. 

**Figure 3:**
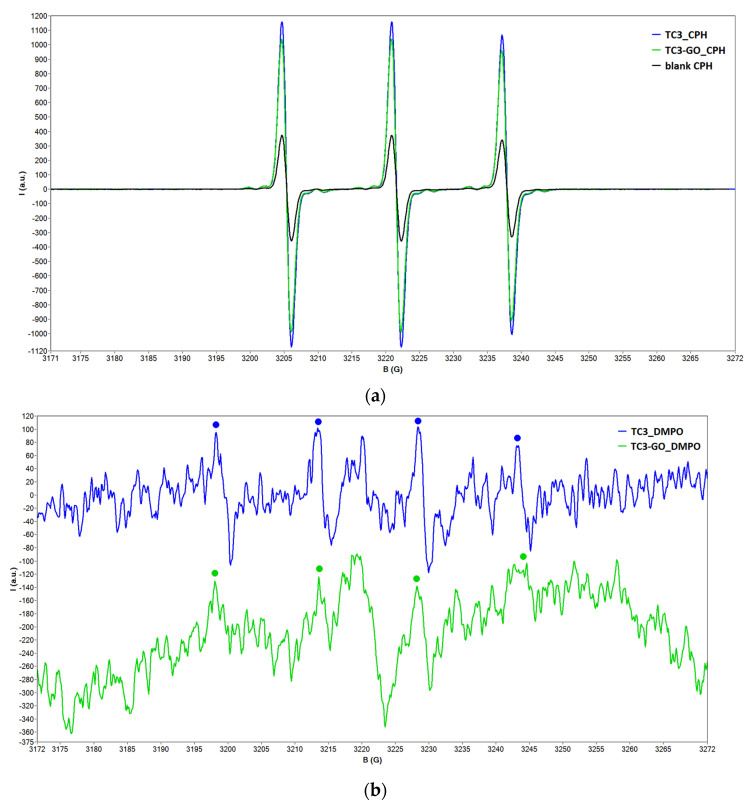
The EPR spectra of the TC3 and TC3-GO samples in the presence of (**a**) CPH and (**b**) DMPO.

The EPR spectra of the TiO_2_ aqueous samples in the presence of spin traps are shown in Figure 3. One may observe the characteristic 1:1:1 triplet signal of the stable 3-carboxy-carproxyl nitroxide formed by oxidation of CPH by ROS [25] (Figure 3a) and the 1:2:2:1 quartet signal characteristic to the ^•^DMPO-OH spin adduct [26] (Figure 3b). The hyperfine coupling constant of the stable nitroxide was determined from the experimental spectrum as a_N_ = 16.18 G, typical for a nitroxide. The hyperfine coupling constants of the ^•^DMPO-OH spin adduct, obtained by spectral simulation, are a_N_ = 15.00 G and a_H_^β^ = 14.56 G, in accordance to the data reported in ref. [27]. Signal intensities are slightly lower for the sample containing graphene oxide. The weak signal recorded for the ^•^DMPO-OH spin adduct may indicate a fast consumption of the HO^•^ radical in these systems.

### 2.3. Electrochemical Characterization

#### 2.3.1. Cyclic Voltammetry Study

**Figure 4:**
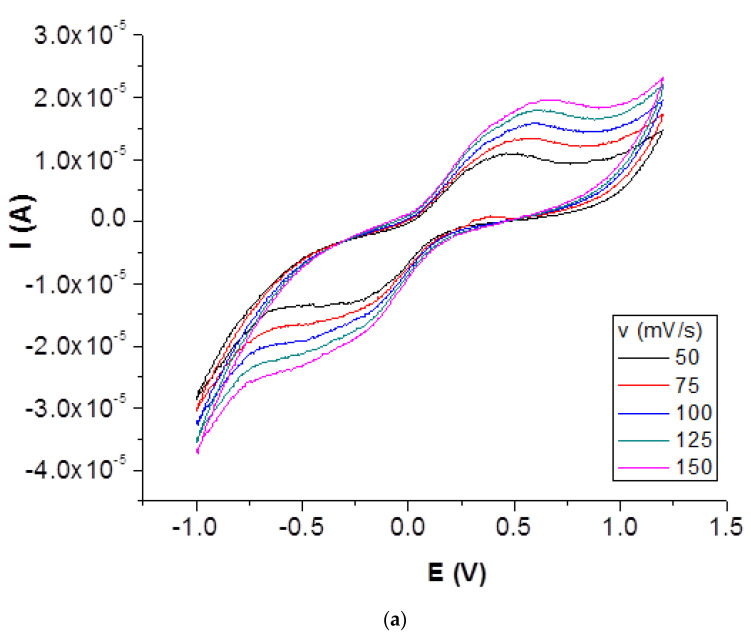

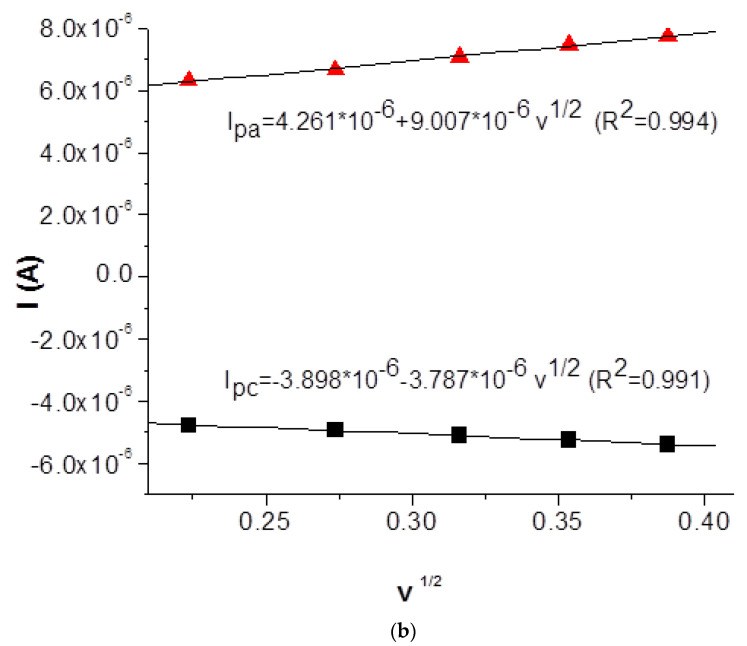
(**a**) Cyclic voltammograms for 1.0 mM K_3_[Fe(CN)_6_] in 0.1 M KCl solution on TC2 modified carbon paste electrode, v = 50–150 mV s^−1^) and (**b**) I vs. v½ plot.

The results show that the anodic peaks increase when the sweep rate is increased and there is a move to positive potentials. Additionally, by increasing the sweep rate, the peak shape does not change, which leads to the conclusion that the modified electrode is sensitive regarding the electrochemical investigation of the ferri/ferro process. In Figure 4a, cyclic voltammograms for TC2 are presented. 

An analysis of the voltammetric peak height as a function of the square-root of the scan rate reveals a highly linear response, with good correlation factors, as observed in Figure 4b. This response indicates a diffusion-controlled electrochemical process.

**Figure 5:**
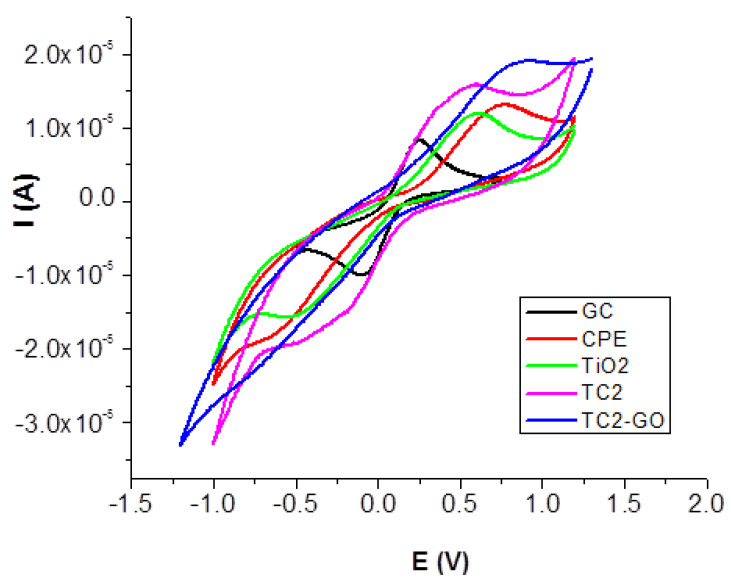
Overlay of the cyclic voltammograms for selected TC2 modified electrodes for the redox process of 1.0 mM K_3_[Fe(CN)_6_] in 0.1 M KCl solution (scan rate was 100 mV s^−1^).

The same experiments were performed for all modified electrodes. A comparison of CV measurements at 100 mV/s for all electrodes is presented in Figure 5.

Electrochemical CV data for bare and modified carbon-paste electrodes are presented in Table 2.

Both anodic and cathodic peak potentials are shifted for TiO_2_ and TC2 when compared with the potential of bare carbon paste electrode. An increase in the peak currents and a decrease in the separation between the peak potentials (ΔEp) at 100 mV·s^−1^ were observed for these two modified electrodes in comparison to the bare CPE, indicating that the electron transfer reaction was kinetically and thermodynamically favored at the copper oxides-decorated TiO_2_-modified electrode surface. Enhanced electron transfer capacity was also found by CV by Mirza-Aghayan et al. for the CuO/rGO/TiO2 system [12]. In the case of the electrode modified with copper oxide-decorated TiO_2_/graphene oxide, peak currents and potentials decreased. By increasing the scan rate, the intensity of the peak increases not only in the anodic direction but also in the cathodic side. The parameter of most significant importance is represented by the position of the voltammetric peak rather than the magnitude of the wave. In the case of the metal-doped graphene modified electrodes, the larger peak current is likely due to a slightly larger surface area at the electrode.

Large band gap narrowing of Cu_2_O/CuO-decorated TiO_2_/graphene oxide nanocomposites: TC1, 2.90 eV, TC2, 2.94 eV, TC3, 2.86 eV, TC1-GO, 2.75 eV, TC2-GO, 2.56 eV, TC3-GO, and 2.76 eV as compared to pure TiO2, 3.2 eV reported previously [9] may explain their enhanced electron transfer capacity. 

#### 2.3.2. Differential Pulse Voltammetry Results

**Figure 6:**
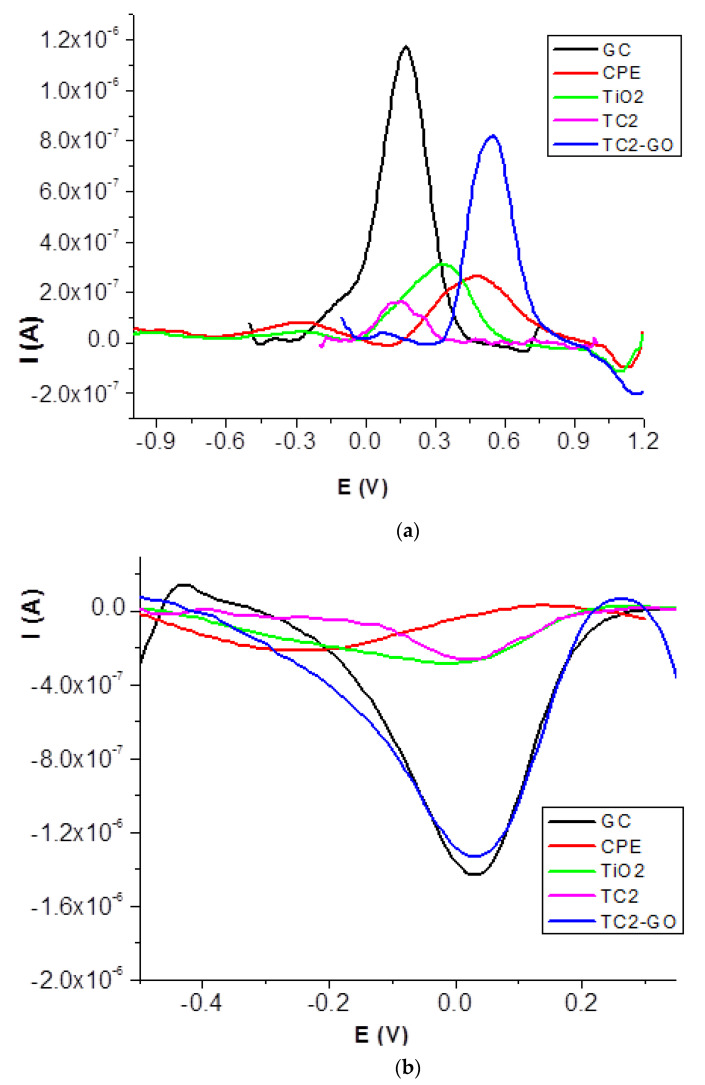
Differential pulse voltammograms for TC2 modified electrodes for the redox process of 1.0 mM K_3_[Fe(CN)_6_] in 0.1 M KCl solution (with step potential 10 mV and modulation amplitude 25 mV): (**a**) anodic and (**b**) cathodic waves.

To get a better understanding of the redox behavior at the modified electrodes, DPV measurements were performed. Differential pulse voltammetry is a more sensitive technique than cyclic voltammetry. The DPV traces are presented in Figure 6.

Anodic peak potentials shifted positively for all carbon paste bare and modified electrodes when compared with glassy carbon (0.175 V). The best response was obtained for the TC2-GO electrode, as stated by the highest peak current values (increased by 2.5 to 5.5). For the reduction process, the peak potential and peak current at TC2-GO (0.031 V and −1.41 × 10^−6^ A) were almost the same as those at glassy carbon (0.035 V and −1.46 × 10^−6^ A). For all other electrodes, the peak potentials shifted cathodically, and the peak currents were 4–5 times smaller. The electrodes investigated may be a useful and cheap alternative for the determination of redox active compounds contained in drug formulae.

Reported scientific data showed that the electrochemical response of graphene modified electrodes can be improved by increasing the amount of graphene in the electrode [28].

## 3. Materials and Methods

### 3.1. Nanomaterials Preparation

Cu_2_O/CuO-decorated TiO_2_/graphene oxide nanocomposites were prepared by the liquid impregnation method as previously described [29]. Briefly, TiO_2_ powder (P25 Evonik) was dispersed in the appropriate solutions of copper (II) nitrate trihydrate under magnetic stirring. Subsequently, the dispersing medium was evaporated, and the resulting powders were calcinated in argon atmosphere at 450 °C, then in argon/hydrogen (10% H_2_) at 280 °C. For high homogeneity, all three powders were dispersed in double-distilled water, frozen, and freeze dried. The resulting powders were denoted TC1, TC2, and TC3 according to the copper content 1%, 2%, and 3%, respectively. The TC(1,2,3)-graphene oxide nanocomposites were also prepared using the freeze-drying procedure by mixing TC1, TC2, and TC3 powders with graphene oxide (GO) in a weight ratio of 10:1. The final powders were denoted TC1-GO, TC2-GO, and TC3-GO. Graphene oxide was synthesized according to an improved version of Hummer’s method that was reported elsewhere [30].

### 3.2. Photoluminescence and EPR Spectroscopy Characterization

Photoluminescence (PL) spectra of the nanocomposites were recorded using a Jasco FP-6500 spectrofluorimeter equipped with a 150 W Xenon lamp. The excitation wavelength used was 320 nm. The experiments were performed in triplicate.

The EPR spectra of the solid probes were recorded on a JEOL FA100 spectrometer equipped with a cylindrical-type resonator TE011 using the following parameters: frequency modulation 100 kHz, microwave power 0.998 mW, sweep time 1800 s, modulation amplitude 1 G, time constant 1 s, and magnetic field scan range 1500 G. Each solid sample was placed in a glass capillary and introduced in the spectrometer’s cavity. 

For the spin-trapping measurements, two spin traps, 5,5-dimethyl-1-pyrroline N-oxide (DMPO) and 1-hydroxy-3-carboxy-2,2,5,5-tetramethylpyrrolidine (CPH), purchased from Sigma-Aldrich (St. Louis, MO, USA) and ENZO Life Sciences, Inc. (Lausen, Switzerland), respectively, were used. The parameter settings of the EPR spectrometer for spin-trapping experiments were frequency modulation 100 kHz, microwave power 0.998 mW, sweep time 60 s, modulation amplitude 1 G, time constant 0.1 s, and magnetic field scan range 100 G.

Aqueous dispersions containing TiO_2_-based samples (1 mg/mL), DMPO (25 mM), and hydrogen peroxide (125 mM) were prepared and incubated for 10 min at 37 °C in the dark. The samples were vortexed during the last 5 min of incubation and then centrifuged at 15.000 rpm for 1 min. The supernatant was immediately transferred into a capillary tube and exposed for 10 min to UVA radiation (370 nm, mercury arc lamp, 500 W, LOT-Quantum Design, Darmstadt, Germany) directly in the spectrometer’s cavity; then, the EPR spectrum was recorded.

A stock solution of CPH (10 mM) was prepared in phosphate buffer of pH 7.4. To this solution, the chelating agent deferoxamine mesylate (100 μM) was added in order to prevent the oxidation of CPH that is catalyzed by traces of transition metal ions [31]. Aqueous dispersions containing TiO_2_ (1 mg/mL) and CPH (0.5 mM) were prepared and treated similarly to the case of the DMPO-containing samples. The experiments were performed in duplicate.

The simulation of the EPR spectra of spin adducts formed by the DMPO and CPH spin traps with the ROS generated by TiO_2_ samples was performed using the WinSim program [32,33].

### 3.3. Preparation of the Carbon Paste Electrodes

Graphite powder (GP) (<20 µm, synthetic, Sigma-Aldrich), mineral oil (MO) (Sigma-Aldrich), and copper oxides-TiO_2_ graphene oxide powders were used for the preparation of the electrodes. A certain amount of graphite powder was placed in a mortar and pestle and was thoroughly hand mixed for 40 min with paraffin oil, until a consistent uniformly wetted paste was obtained. The GP:MO ratio was approximately 3:1 (*w*/*w*). The obtained paste was placed into a plastic syringe of 1.0 mL. The electrical contact was made by forcing a copper wire down into the syringe and into the back of the graphite paste. The surface of the electrodes was obtained by polishing it on a weighing paper and, when it was necessary, a renewed surface was made by pushing a small excess of the paste out of the tube and polishing it again. The bare carbon paste electrode will be denoted by CPE. The modified electrodes were prepared by mixing certain amounts of carbon paste with Cu_2_O/CuO-decorated TiO_2_ graphene oxide nanocomposites (97:3 *w*/*w*) and were denoted by TC1, TC2, TC3, TC1-GO, TC2-GO, and TC3-GO. The obtained materials were pressed at the end of carbon paste from syringes. For comparison, a few electrodes were also prepared as follows: one was left unmodified (CPE), one modified with graphene oxide (denoted by GO), and one modified with TiO_2_ powder (denoted TiO_2_). The surface of all electrodes was smoothed by polishing on a piece of weighing paper. All electrodes were kept in distilled water before and after measurements.

Electrochemical measurements were carried out in duplicate on a potentiostat galvanostat system AutoLabPGStat 12, controlled by a general purpose electrochemical system (GPES) with interface for Windows (version 4.9.007). Three electrodes in a one compartment cell (10 mL) were used in all experiments. A glassy carbon electrode (Metrohm, 3 mm in diameter) and carbon-paste electrodes (unmodified and modified) served as working electrodes. The counter electrode was a Pt wire of large area. All experimental potentials were referred to Ag/AgCl/KClsat as reference electrodes. 

### 3.4. Testing the Modified Carbon Paste Electrodes

The electrochemical characterization of the modified CPE electrodes was carried out by cyclic voltammetry (CV) and differential pulse voltammetry (DPV). The CV experiments were recorded in 0.1 mol L^−1^ KCl solution containing 1.0 mmol L^−1^ K_3_[Fe(CN)_6_] in the potential range of (−1) to (+1.2) V at scan rates of 50 to 150 mV s^−1^. DPV curves were recorded on the same potential domains at step potential (SP) 10 mV and modulation amplitude (MA) 25 mV.

All modified electrodes were tested for the redox process of 1 mM potassium ferrocyanide(III) using 0.1 M KCl as electrolyte, being an one-electron reversible redox system.

[Fe(CN)_6_] ^3−^  +  e^−^ ↔ [Fe(CN)_6_] ^4−^

A control experiment was first performed utilizing a bare carbon paste electrode. The voltammetric profile of bare CPE and copper oxides-TiO_2_ ± graphene oxide-modified electrodes was explored by sweep rate variation from 50 to 150 mV/s.

## 4. Conclusions

The enhanced free radical generation and electrochemical response of Cu_2_O/CuO-decorated TiO_2_/graphene oxide nanocomposites are associated with modifications of transition metal oxides. Their electron-accepting properties may enhance the oxidation of analyte when anodic scans are performed. The potential application of the demonstrated electrochemical properties of modified electrodes with nanostructured oxides may increase the efficiency of drug detection in electroanalysis through electro-catalytic effects. Additionally, it was shown for the first time that the obtained modified TiO_2_ nanocomposites transfer electrons under UV irradiation and generate hydroxyl and superoxide radicals reactive oxygen species (ROS), as emphasized by EPR spectroscopy. Further applications of these new nanomaterials could be bacterial inactivation; obtaining self-cleaning surfaces; sensors; and various uses in environmental remediation.

## Figures and Tables

**Table 1:** The g factors of the solid samples investigated.

Sample	ν (GHz)	B (mT)	g
TC1	9.046638	300.419	2.1516
TC1-GO	9.047555	299.914 310.639	2.1554 2.0810
TC2	9.046588	301.141	2.1464
TC2-GO	9.047630	298.793 310.079	2.1635 2.0848
TC3	9.047059	300.865	2.1485
TC3-GO	9.046954	298.233 309.038	2.1674 2.0916

**Table 2:** Electrochemical data from CV measurements at 100 mV/s; I_a_ and I_c_ represent the anodic and cathodic peak currents, and E_a_ and E_c_ represent the anodic and cathodic peak potentials.

	E_c_ (V)	I_c_ (A)	E_a_ (V)	I_a_ (A)	ΔE (V)
CPE	−0.568	−2.69 × 10^−6^	0.706	6.22 × 10^−6^	1.274
TiO_2_	−0.424	−4.69 × 10^−6^	0.567	7.28 × 10^−6^	0.991
TC2	−0.203	−5.00 × 10^−6^	0.466	7.08 × 10^−6^	0.669
TC2-GO	−0.557	−1.07 × 10^−6^	0.768	6.04 × 10^−6^	1.325

## Data Availability

Data are available on request from the authors.
